# Analysis of Time to Treatment and Survival Among Adults Younger Than 50 Years of Age With Colorectal Cancer in Canada

**DOI:** 10.1001/jamanetworkopen.2023.27109

**Published:** 2023-08-03

**Authors:** Matthew Castelo, Lawrence Paszat, Bettina E. Hansen, Adena S. Scheer, Neil Faught, Lena Nguyen, Nancy N. Baxter

**Affiliations:** 1 Department of Surgery, University of Toronto, Toronto, Ontario, Canada; 2 Institute of Health Policy, Management and Evaluation, Dalla Lana School of Public Health, University of Toronto, Toronto, Ontario, Canada; 3 ICES, Toronto, Ontario, Canada; 4 Department of Epidemiology, Erasmus MC, Rotterdam, the Netherlands; 5 Li Ka Shing Knowledge Institute, St Michael’s Hospital, Toronto, Ontario, Canada; 6 School of Population and Global Health, University of Melbourne, Melbourne, Victoria, Australia

## Abstract

**Importance:**

Colorectal cancer (CRC) is uncommon in adults younger than 50 years of age, so this population may experience delays to treatment that contribute to advanced stage and poor survival.

**Objective:**

To investigate whether there is an association between time from presentation to treatment and survival in younger adults with CRC.

**Design, Setting, and Participants:**

This retrospective cohort study used linked population-based data in Ontario, Canada. Participants included patients with CRC aged younger than 50 years who were diagnosed in Ontario between 2007 and 2018. Analysis was performed between December 2019 and December 2022.

**Exposure:**

Administrative and billing codes were used to identify the number of days between the date of first presentation and treatment initiation (overall interval).

**Main Outcomes and Measures:**

The associations between increasing overall interval, overall survival (OS), and cause-specific survival (CSS) were explored with restricted cubic spline regression. Multivariable Cox proportional hazards models were also fit for OS and CSS, adjusted for confounders. Analyses were repeated in a subset of patients with lower urgency, defined as those who did not present emergently, did not have metastatic disease, did not have cross-sectional imaging or endoscopy within 14 days of first presentation, and had an overall interval of at least 28 days duration.

**Results:**

Among 5026 patients included, the median (IQR) age was 44.0 years (40.0-47.0 years); 2412 (48.0%) were female; 1266 (25.2%) had metastatic disease and 1570 (31.2%) had rectal cancer. The lower-urgency subset consisted of 2548 patients. The median (IQR) overall interval was 108 days (55-214 days) (15.4 weeks [7.9-30.6 weeks]). Patients with metastatic CRC had shorter median (IQR) overall intervals (83 days [39-183 days]) compared with those with less advanced disease. Five-year overall survival was 69.8% (95% CI, 68.4%-71.1%). Spline regression showed younger patients with shorter overall intervals (<108 days) had worse OS and CSS with no significant adverse outcomes of longer overall intervals. In adjusted Cox models, overall intervals longer than 18 weeks were not associated with significantly worse OS or CSS compared with those waiting 12 to 18 weeks (OS: HR, 0.83 [95% CI, 0.67-1.03]; CSS: HR, 0.90 [95% CI, 0.69-1.18]). Results were similar in the subset of lower-urgency patients, and when stratified by stage.

**Conclusions and relevance:**

In this cohort study of 5026 patients with CRC aged younger than 50 years of age in Ontario, time from presentation to treatment was not associated with advanced disease or poor survival. These results suggest that targeting postpresentation intervals may not translate to improved outcomes on a population level.

## Key Points

**Question:**

Is longer time from presentation to treatment start associated with worse survival in patients with colorectal cancer younger than 50 years of age?

**Findings:**

In this cohort study including 5026 patients with colorectal cancer aged younger than 50 years diagnosed in Ontario, Canada, between 2007 and 2018, time between first presentation and treatment start was calculated. Longer time to treatment was not associated with adverse overall or cause-specific survival, and similar results were seen in a subset of patients with lower-urgency presentations.

**Meaning:**

These findings suggest that postpresentation delays in younger adults with colorectal cancer do not appear to be associated with worse outcomes.

## Teaser

This cohort study investigates whether there is an association between time from presentation to treatment and survival in younger Canadian adults with colorectal cancer.

## Introduction

The incidence of colorectal cancer (CRC) among adults younger than 50 years of age is rising globally.^1^ This population is also more likely to present with metastatic disease compared with older adults^2^ and has worse survival.^3,4^ However, the reasons for worse outcomes in young adults is unclear.^5,6^ The majority of younger adults have sporadic CRC, are not eligible for screening, and therefore present symptomatically.^7^ Several authors have suggested lack of screening, low suspicion of CRC from physicians, and poor access to care leading to excessively delayed treatment could be contributory.^5,7,8^

To evaluate delay in this population, we previously examined the time from first presentation to treatment initiation^9^; adults younger than 50 years of age in Ontario, Canada, waited a median of 109 days. One-quarter of patients waited longer than 218 days.^9^ Given that delay is proposed as a potential mechanism for worse outcomes in young people with CRC, there is insufficient understanding of the relationship between time to treatment (or other intervals) and survival in these patients. The literature specifically examining adults younger than age 50 years is sparse. In a systematic review of 55 studies^10^ reporting delay intervals in this population, only 2 examined survival in young people and 1 found worse outcomes with longer delay.^11,12^ These 2 studies have limitations; the first^12^ is a single-center analysis, and the second^11^ did not assess survival beyond 1 year.

Although the association between delay and survival has been explored in older patient populations, it is challenging to extrapolate to younger patients because they have unique clinicopathologic characteristics and pathways to treatment.^13,14,15,16,17^ Because delay in this population may be an important target for intervention, exploring the association between delay intervals and outcomes in younger adults with CRC is timely. The aim of this study was to evaluate the association between the interval from presentation to treatment initiation with overall and cause-specific survival in adults younger than 50 years of age with CRC using high-quality, population-based data sets in Ontario, Canada.

## Methods

### Study Design and Data Sources

This was a population-based cohort study. Data were obtained from ICES, an independent, nonprofit research institute that maintains health administrative data for more than 14 million Ontario residents. These data sets were linked using unique encoded identifiers and analyzed at ICES (eTable 1 in Supplement 1). We followed the Strengthening the Reporting of Observational Studies in Epidemiology (STROBE) reporting guideline.^18^ The research ethics board at St Michael’s Hospital approved this study and informed consent was not required, in accordance with section 45 of Ontario’s Personal Health Information Protection Act.

### Patient Population

We identified Ontario residents aged 15 to 49 years who were diagnosed with CRC between January 1, 2007, and December 31, 2018, using the Ontario Cancer Registry (OCR). CRC stage is only available after 2007 in the OCR. Exclusion criteria included death on or before diagnosis date, atypical histology, inflammatory bowel disease, missing sex or stage, inability to assign a date of first presentation or treatment, and time from presentation to treatment greater than 18 months (eFigure 1 in Supplement 1).

The subset of patients with lower urgency were defined as those with stage I to III disease who did not present emergently, did not receive computed tomography (CT), magnetic resonance imaging (MRI), or lower endoscopy within 14 days after first presentation, and had an overall interval (time from presentation to treatment) of at least 30 days duration. Guidelines targeted at primary care physicians in Canada for the workup and referral of symptomatic patients at risk of CRC recommend urgent consultation and initiation of workup (within 2 weeks of presentation) for those with highly suspicious findings (eg, palpable rectal mass).^19^

### Exposure

Our exposure of interest was the number of days from first presentation to treatment initiation (the overall interval). To identify the date of first presentation, we used an algorithm based on administrative and billing codes, adapted from prior work by Groome et al^20,21,22,23^ for CRC, breast cancer, and others. We have described our application of these methods to measure delay intervals among 6853 Ontario adults younger than age 50 years with CRC, and we were able to assign a date of presentation to more than 97% of the cohort.^9^ Briefly, we searched for the earliest clinical encounter for CRC-related signs or symptoms, up to a maximum of 18 months prior to diagnosis. Relevant clinical encounters were defined by groups of administrative and billing codes representing symptoms, diagnostic tests, diagnostic procedures, and surgical procedures.

The date of diagnosis was identified from the OCR. The date of treatment initiation was defined as the first date of radiotherapy, chemotherapy, or surgery after diagnosis.

### Outcomes

Outcomes included overall survival (OS) and cause-specific survival (CSS). OS was defined as the number of months from treatment start to death, or until December 31, 2019. Deaths for cause-specific survival were classified as death due to CRC, or death due to other causes. CSS was defined as the number of months from treatment start to death due to CRC, or until December 31, 2019. Patients were censored if they experienced a non-CRC death.

### Covariates

Age at diagnosis and sex were determined from the Registered Persons Database.^24^ Marginalization was measured using the Ontario Marginalization Index (ON-Marg).^25^ We used the Johns Hopkins Adjusted Clinical Group system to identify Major Aggregated Diagnosis Groups (ADGs).^26,27^ Emergency presentations were defined as a first presentation occurring in the context of an emergency department visit or hospital admission, or preceding hospitalization within 3 days. Using administrative codes on the date of first presentation, symptomatology was categorized as anemia, gastrointestinal (GI) symptoms, or none/not determined. Based on the first type of imaging performed after the first date of presentation, initial imaging was divided into cross-sectional (CT or MRI), non–cross-sectional (abdominal ultrasound or x-ray), or no abdominal imaging. Detailed covariate definitions are presented in eTable 2 in Supplement 1.

Disease characteristics, including cancer site, stage, and tumor histology were obtained from the OCR. Histology was categorized as adenocarcinoma or no special type, mucinous adenocarcinoma, and other.

### Statistical Analysis

Patient characteristics were described for the entire cohort and a subset of patients with lower-urgency pathways. Differences between age groups were compared using Wilcoxon rank-sum tests for continuous variables and χ^2^ tests for categorical variables.

We generated cumulative incidence curves for CRC deaths and non-CRC deaths for the cohort. The association between longer overall intervals and survival (OS and CSS) was explored using univariate restricted cubic spline regression and 3 knots at equally spaced percentiles. The effect size was centered at the median overall interval for the cohort, and hazard ratios (HRs) and 95% CIs presented. Splines were fit in the overall cohort and stratified by stage of disease.

The associations of longer overall intervals with OS and CSS were tested using Cox proportional hazards models. The overall interval was divided into 6-week increments. The reference group was set at 12 to 18 weeks because it was a typical interval from presentation to treatment start for patients in whom there is reasonable suspicion of CRC. The Canadian Association of Gastroenterology Wait Time Consensus Group recommends the maximum wait time to lower endoscopy for patients with iron-deficiency anemia, change in bowel habits, bright red blood per rectum, or a positive stool test is 8 weeks.^28^ After diagnosis, standards set in Ontario propose that patients with CRC see a surgeon within 21 days and receive surgery thereafter within 28 days.^29^ Thus, the overall recommended time from presentation and referral to endoscopy and subsequent treatment is approximately 15 weeks.

Cox models were adjusted for sex, age, number of major ADGs, symptomatology, ON-Marg score, emergency presentation, and cancer site. The aforementioned analyses were also repeated for lower-urgency subsets. We performed sensitivity analyses, running the models stratified by stage and including patients with metastatic disease in the lower-urgency subset.

Missing data were handled using pairwise deletion.^30^ The analysis was performed using SAS version 9.4 (SAS Institute) and R version 4.1.0 (R Foundation for Statistical Computing) between December 2019 and December 2022. All statistical tests were 2-sided, and *P* ≤ .05 was considered statistically significant.

## Results

### Patient and Disease Characteristics

Among the 5026 adults included in the analysis (eFigure 1 in Supplement 1), the median (IQR) age was 44.0 years (40.0-47.0 years) and 2412 (48.0%) were female; 2380 (47.4%) had no major comorbidity and 1670 (33.2%) had a single major comorbidity; 1266 (25.2%) presented with metastatic disease and 1570 (31.2%) had rectal cancer (Table 1). The lower-urgency subset consisted of 2548 patients (Table 1). Their demographic characteristics were similar to the remaining 2501 patients. Patients in the lower-urgency subset were less likely to receive cross-sectional imaging before diagnosis (424 [16.6%] vs 741 [29.1%]; *P* < .001) and were more likely to have rectal cancer (894 [35.1%] vs 676 [26.5%]; *P* < .001) compared with the remaining patients.

**Table 1.** Characteristics for the Cohort of Patients With Colorectal Cancer, Stratified by Urgency

Characteristic	Patients, No.			*P* value^b^
	Entire cohort (n = 5026)	Subset of patients with lower urgency (n = 2525)^a^	Remaining cohort (n = 2501)	
Age, median (IQR), y	44.0 (40.0-47.0)	45.0 (41.0-47.0)	44.0 (39.0-47.0)	<.001
Sex				
Male	2614 (52.0)	1270 (50.3)	1344 (53.7)	.02
Female	2412 (48.0)	1255 (49.7)	1157 (46.3)	
No. of major ADGs				
0	2380 (47.4)	1265 (50.1)	1115 (44.6)	<.001
1	1670 (33.2)	829 (32.8)	841 (33.6)	
2	704 (14.0)	321 (12.7)	383 (15.3)	
≥3	272 (5.4)	110 (4.4)	162 (6.5)	
ON-Marg Summary Score^c^	3.00 (2.25-3.50)	2.75 (2.25-3.50)	3.00 (2.50-3.50)	.009
Missing	50	14	36	NA
Emergency presentation	1209 (24.1)	0	1209 (48.3)	<.001
Symptomatology				
Anemia	336 (6.7)	200 (7.9)	136 (5.4)	<.001
Gastrointestinal symptoms	4521 (90.0)	2254 (89.3)	2267 (90.6)	
None/not determined	169 (3.4)	71 (2.8)	98 (3.9)	
Imaging before diagnosis				
Cross-sectional	1165 (23.2)	424 (16.8)	741 (29.6)	<.001
No abdominal/CRC-directed imaging	1997 (39.7)	1249 (49.5)	748 (29.9)	
Non–cross-sectional	1864 (37.1)	852 (33.7)	1012 (40.5)	
Stage				
I	755 (15.0)	567 (22.5)	188 (7.5)	<.001
II	1076 (21.4)	694 (27.5)	382 (15.3)	
III	1929 (38.4)	1264 (50.1)	665 (26.6)	
IV	1266 (25.2)	0	1266 (50.6)	
Histology				
Adenocarcinoma or no special type	4571 (90.9)	2308 (91.4)	2263 (90.5)	.04
Mucinous adenocarcinoma	334 (6.6)	170 (6.7)	164 (6.6)	
Other	121 (2.4)	47 (1.9)	74 (3.0)	
Disease site				
Proximal colon	1713 (34.1)	762 (30.2)	951 (38.0)	<.001
Sigmoid and rectosigmoid	1743 (34.7)	869 (34.4)	874 (34.9)	
Rectum	1570 (31.2)	894 (35.4)	676 (27.0)	
Overall interval, d				
Median (IQR)	108 (55-214)	142 (86-248)	71 (31-168)	<.001
Mean (SD)	146 (119)	177 (114)	116 (116)	
Range	0-545	28-545	0-525	

Abbreviations: ADGs, Aggregated Diagnosis Groups; CRC, colorectal cancer; NA, not applicable; ON-Marg, Ontario Marginalization Index.

^a^ Subset of patients with lower urgency was defined as those who did not present emergently, did not have metastatic disease, did not have cross-sectional imaging or endoscopy within 14 days of first presentation, and had an overall interval of at least 28 days duration.

^b^ *P* values calculated using Wilcoxon rank-sum test or Pearson χ^2^ test.

^c^ The ON-Marg ranges from 1 to 5; higher values indicate a higher degree of marginalization.

### Time From Presentation to Treatment Start (Overall Interval)

**Figure 1.**
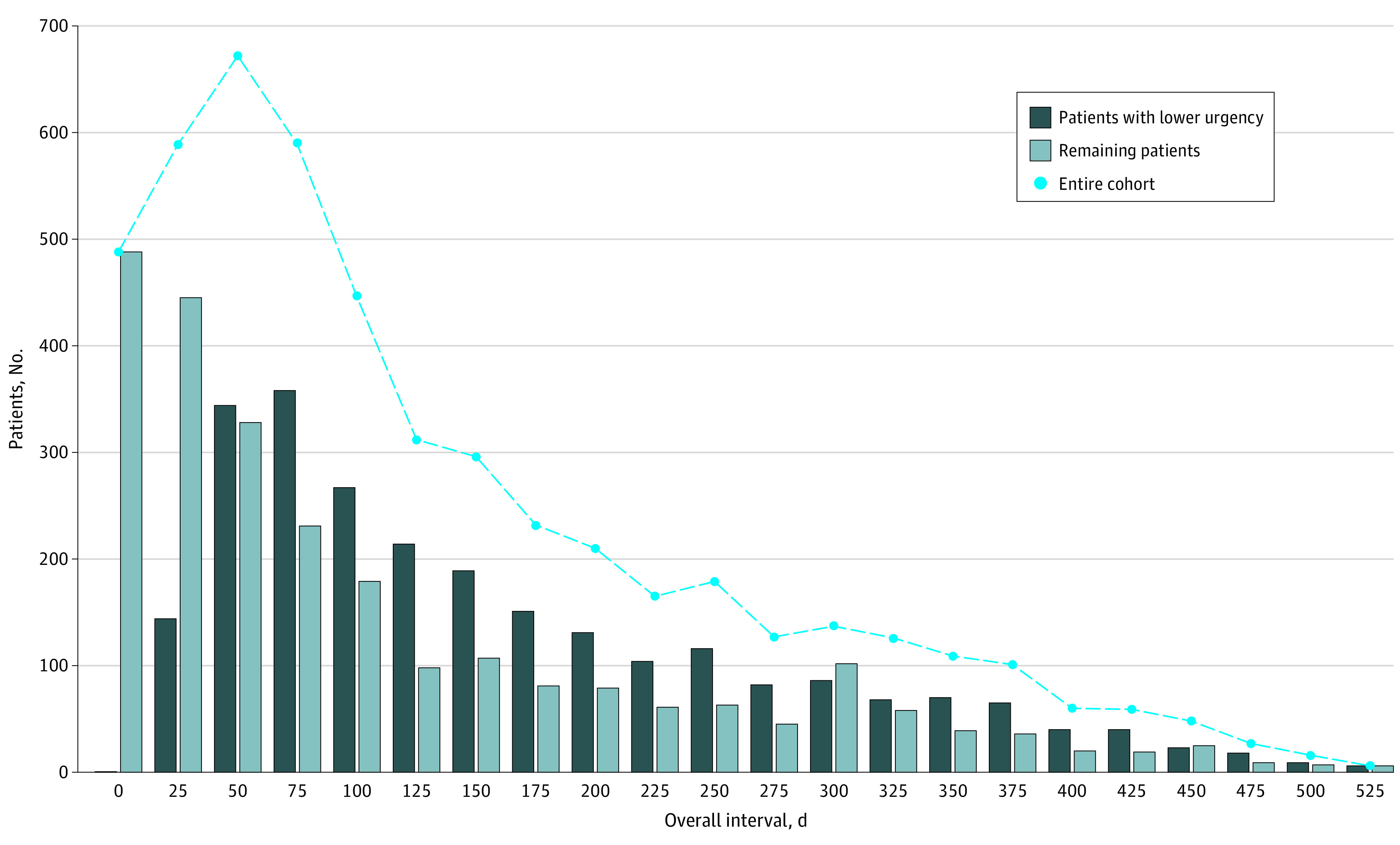
Distribution of Time From Presentation to Treatment Start (Overall Interval) for Subset of Patients With Lower-Urgency Colorectal Cancer and the Remaining Cohort

The median (IQR) overall interval for the cohort was 108 days (55-214 days) (15.4 weeks [7.9-30.6 weeks]). For the lower-urgency subset, the median (IQR) overall interval was longer at 141 days (85-246 days) (20.1 weeks [12.1-35.1 weeks]), a difference that was statistically significant (*P* < .001) (Table 1). The distribution for the overall interval by urgency is shown in Figure 1.

Stage was associated with the length of the overall interval. Patients with stage I CRC waited a median (IQR) 152 days (87-261 days) to treatment, compared with a median (IQR) 108 days (54-217 days) for those with stage II, and a median (IQR) 107 days (58-210 days) for those with stage III CRC. Patients with metastatic CRC had the shortest median (IQR) overall intervals (83 days [39-183 days]) (eFigure 2 in Supplement 1).

### Overall Interval and Survival

Over the study period, 1574 patients died (31.3%). Of these, 1041 died due to CRC (66.1% of deaths). The 5-year OS was 69.8% (95% CI, 68.4%-71.1%) and 10-year OS was 63.0% (95% CI, 61.5%-64.6%). The 5-year CSS was 78.2% (95% CI, 77.0%-79.4%) and 10-year CSS was 75.0% (95% CI 73.7%-76.4%). The cumulative incidences of CRC and non-CRC deaths are shown in eFigure 3 in Supplement 1. Survival decreased with advancing stage: 5-year OS for patients with stage I CRC was 95.5% (95% CI, 93.9%-97.1%), falling to 20.5% (95% CI, 18.0%-23.0%) for those with metastatic disease (eFigure 3 in Supplement 1). Similar patterns were seen for CSS.

**Figure 2.**
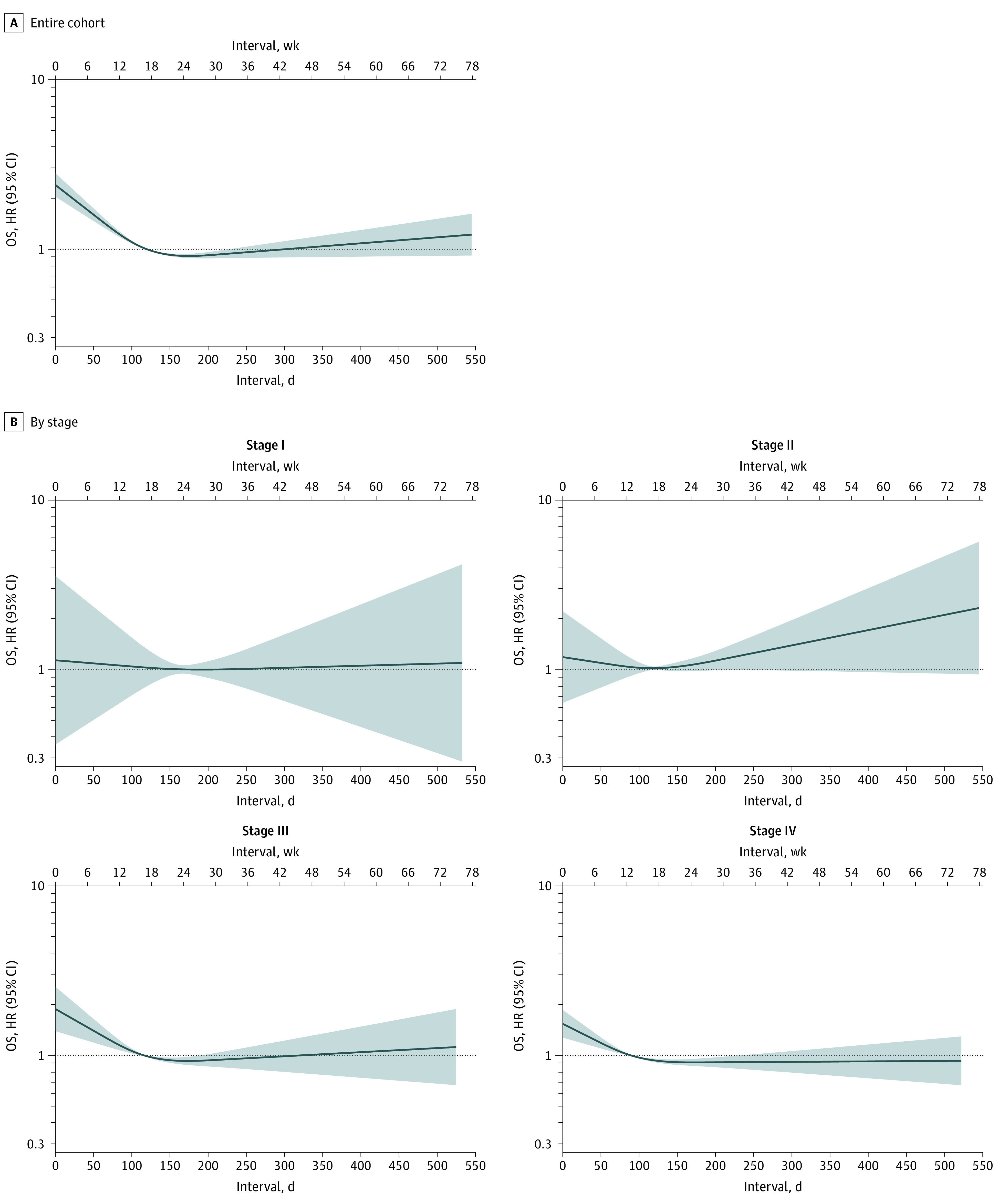
Restricted Cubic Spline Regression Showing Univariate Association of Increasing Time From Presentation to Treatment (Overall Interval) With Overall Survival (OS) Figure shows restricted cubic spline regression data for entire cohort (A) and stratified by stage of colorectal cancer (B). Hazard ratios (HRs) shown on a log scale; shaded areas indicate 95% CIs.

Using spline regression, younger adults with overall intervals shorter than the median (<108 days) had worse OS, reflecting the predilection for patients with metastatic disease and urgent presentations to have shorter times to treatment (Figure 2A). Those with longer times to treatment had similar OS to those with median overall intervals (108 days). Longer overall intervals were not associated with adverse survival in spline regression when examining CSS (eFigure 4 in Supplement 1). When stratified by stage (Figure 2B), increasing overall interval was not associated with significantly worse OS. In stratified analyses, adverse survival was not seen for patients with stage I and II cancer with short overall intervals.

In adjusted Cox proportional hazards models including all patients (Table 2), overall interval lengths less than 6 weeks and 6 to 12 weeks were associated with worse outcomes compared with interval lengths of 12 to 18 weeks for both OS (<6 weeks: HR, 1.73 [95% CI, 1.46-2.06]; 6-12 weeks: HR, 1.21 [95% CI, 1.03-1.43]) and CSS (<6 weeks: HR, 2.06 [95% CI, 1.67-2.55]; 6-12 weeks: HR, 1.31 [95% CI, 1.06-1.61]). Overall intervals longer than 18 weeks were not associated with significantly worse OS or CSS compared with those waiting 12 to 18 weeks. We additionally ran adjusted models stratified by stage of disease which did not show significantly worse OS or CSS with increasing overall interval lengths (eTable 3 in Supplement 1).

**Table 2.** Adjusted Cox Proportional Hazards Model Showing Outcome of Increasing Overall Interval Associated With Overall and Cause-Specific Survival in Entire Patient Cohort

Covariate	Overall survival	Cause-specific survival
Length of overall interval		
0 to <6 wk (n = 858)	1.73 (1.46-2.06)	2.06 (1.67-2.55)
6 to <12 wk (n = 1107)	1.21 (1.03-1.43)	1.31 (1.06-1.61)
12 to <18 wk (n = 851)	1 [Reference]	1 [Reference]
18 to <24 wk (n = 523)	0.83 (0.67-1.03)	0.90 (0.69-1.18)
24 to <30 wk (n = 388)	0.84 (0.66-1.06)	0.94 (0.70-1.26)
30 to <36 wk (n = 313)	0.93 (0.72-1.19)	0.98 (0.72-1.35)
≥36 wk (n = 986)	0.89 (0.75-1.07)	0.90 (0.72-1.14)
Age (increase in 5 y)	0.98 (0.94-1.02)	0.98 (0.94-1.04)
Sex		
Male	1 [Reference]	1 [Reference]
Female	1.02 (0.92-1.13)	1.03 (0.91-1.17)
ON-Marg Summary Score (increase in 1 point)	1.03 (0.97-1.10)	1.02 (0.94-1.11)
No. of major ADGs		
0	1 [Reference]	1 [Reference]
1	1.23 (1.10-1.38)	1.24 (1.08-1.43)
2	1.32 (1.14-1.53)	1.34 (1.11-1.60)
≥3	1.56 (1.27-1.92)	1.16 (0.87-1.55)
Anemia	0.92 (0.75-1.14)	0.87 (0.67-1.14)
Symptomatology		
Gastrointestinal symptoms	1 [Reference]	1 [Reference]
None/not determined	1.57 (1.24-1.99)	1.66 (1.25-2.21)
Anemia	0.92 (0.75-1.14)	0.87 (0.67-1.14)
Emergency presentation	1.27 (1.13-1.43)	1.29 (1.11-1.49)
Disease site		
Proximal colon	1 [Reference]	1 [Reference]
Sigmoid and rectosigmoid	1.03 (0.91-1.16)	1.02 (0.88-1.18)
Rectum	0.90 (0.79-1.03)	0.80 (0.69-0.94)

Abbreviations: ADGs, Aggregated Diagnosis Groups; ON-Marg, Ontario Marginalization Index.

### Overall Interval and Survival for Subset of Patients With Lower Urgency

For the lower-urgency subset of patients (N = 2548), spline regression showed worse OS among lower-urgency patients with shorter overall intervals, and no significantly increased OS or CSS for those with longer overall intervals (eFigure 5 in Supplement 1). Multivariable Cox models in this subset reached similar conclusions to the analysis in the overall cohort (Table 3). Overall interval lengths of 18 to 24 weeks and 24 to 30 weeks were associated with similar outcomes compared with interval lengths of 12 to 18 weeks for both OS (18-24 weeks: HR, 0.90 [95% CI, 0.63-1.28]; 24-30 weeks: HR, 0.83 [95% CI, 0.56-1.24]) and CSS (18-24 weeks: HR, 0.85 [95% CI, 0.53-1.36]; 24-30 weeks: HR, 1.08 [95% CI, 0.66-1.75]).

**Table 3.** Unadjusted and Adjusted Cox Proportional Hazards Model Showing Outcome of Increasing Overall Interval Associated With Overall and Cause-Specific Survival in Subset of Patients With Lower Urgency^a^

Covariate	Overall survival	Cause-specific survival
Length of overall interval		
4 to <12 wk (n = 582)	1.00 (0.74-1.36)	0.99 (0.66-1.47)
12 to <18 wk (n = 531)	1 [Reference]	1 [Reference]
18 to <24 wk (n = 351)	0.90 (0.63-1.28)	0.85 (0.53-1.36)
24 to <30 wk (n = 251)	0.83 (0.56-1.24)	1.08 (0.66-1.75)
30 to <36 wk (n = 196)	0.85 (0.55-1.32)	0.88 (0.50-1.56)
≥36 wk (n = 614)	0.86 (0.63-1.16)	0.86 (0.58-1.29)
Age (increase in 5 y)	1.01 (0.92-1.11)	1.02 (0.90-1.15)
Sex		
Male	1 [Reference]	1 [Reference]
Female	0.88 (0.72-1.08)	0.91 (0.69-1.19)
ON-Marg Summary Score (increase in 1 point)	1.01 (0.89-1.16)	0.92 (0.77-1.10)
No. of major ADGs		
0	1 [Reference]	1 [Reference]
1	1.24 (0.98-1.56)	1.07 (0.79-1.45)
2	1.57 (1.16-2.12)	1.43 (0.97-2.11)
≥3	1.74 (1.12-2.71)	1.21 (0.63-2.33)
Symptomatology		
Gastrointestinal symptoms	1 [Reference]	1 [Reference]
None/not determined	0.80 (0.41-1.55)	0.92 (0.41-2.07)
Anemia	1.05 (0.71-1.56)	1.18 (0.72-1.91)
Disease site		
Proximal colon	1 [Reference]	1 [Reference]
Sigmoid and rectosigmoid	1.02 (0.78-1.33)	0.93 (0.65-1.32)
Rectum	1.29 (1.00-1.66)	1.17 (0.84-1.63)

Abbreviations: ADGs, Aggregated Diagnosis Groups; ON-Marg, Ontario Marginalization Index.

^a^ Subset of patients with lower urgency was defined as those who did not present emergently, did not have metastatic disease, did not have cross-sectional imaging or endoscopy within 14 days of first presentation, and had an overall interval at least 28 days duration.

## Discussion

This population-based study of 5026 patients with CRC aged younger than 50 years diagnosed in Ontario between 2007 and 2018 did not find significantly adverse OS or CSS with longer times from presentation to treatment (overall interval). Advanced and metastatic disease was strongly associated with shorter time to treatment, likely explaining why younger patients with CRC with overall intervals less than 100 days had worse outcomes, particularly those with intervals less than 6 weeks (overall mortality: HR, 1.73 [95% CI, 1.46-2.06]). Among a subset of 2548 patients with lower-urgency presentations and nonmetastatic disease, the analysis reached similar conclusions: longer time to treatment was not associated with worse OS or CSS.

To our knowledge, there are few studies examining the association between delay intervals and survival specifically in adults younger than 50 years with CRC.^10^ Di Girolamo et al^11^ performed a population-based analysis in the UK, examining cancer waiting time targets and survival, which included 3542 younger patients with CRC (aged 15-44 years). They analyzed 3 delay intervals: referral to specialist consultation, decision to treat until treatment initiation, and referral to treatment. Longer intervals were not associated with survival.^11^ However, all delay intervals were dichotomized (eg, >2 weeks vs ≤2 weeks), and survival was only assessed at 1-year follow-up. Kim et al^12^ performed a single-center analysis including 693 patients with CRC aged 45 years or younger in Korea between 2006 and 2011. Their results showed an interval from symptom onset to diagnosis greater than 3 months was associated with worse CSS compared with less than 1 month (adjusted HR, 2.57 [95% CI, 1.34-4.94]).^12^ However, this study was small, and the time to diagnosis was rapid (mean of 53 days) in comparison with the literature,^10^ and is also unlikely to be applicable to the Ontario setting. Our study greatly adds to this limited literature in younger adults.

In the CRC literature for older patients, there are mixed findings with respect to delay and survival.^13,14,17,22,31,32^ Ramos et al^13,14^ completed 2 systematic reviews composed primarily of older patients with CRC, focused on the association between delay, stage, and survival. In their analysis, more studies reported improved survival with longer delay intervals (4 of 26 studies) rather than worse survival (2 of 26 studies).^13,14^ Therefore, this literature suggests that similar to our results, longer intervals are not clearly associated with adverse outcomes for patients aged greater than 50 years. If the associations between delay and survival were unique for younger adults, this would be potentially actionable. Patients (younger and older) with longer intervals may be more likely to have more indolent disease, explaining the improved outcomes seen in some studies. This paradoxical finding is well-recognized in the cancer delay literature^17,33,34,35^; patients with advanced and/or aggressive disease who experience distressing symptoms appear to present to medical care sooner and are subsequently investigated and treated expeditiously, leading to shorter delay intervals.^17,33,34,35^ Our findings are consistent with this: young patients with CRC with very short overall intervals disproportionately had metastatic disease, emergency presentations, and poor outcomes in our study. However, this association has also been described in some studies as U-shaped, where patients with the shortest and longest delays have higher mortality.^15,36^ Torring et al^36^ reported U-shaped associations between delay and survival in patients with CRC using combined primary care databases from Denmark and the United Kingdom. Patients with the shortest time from symptom onset to diagnosis and those with intervals greater than the 70th percentile had the highest mortality.^36^ However, in this study the median age was 71 years. We performed spline regression in a similar manner specifically to assess for this pattern among younger adults and did not observe a U-shaped association between delay and survival.

Observational studies examining the prognostic outcome of delay in cancer have implications for policy, as they provide the primary evidence base for efforts to monitor wait times, and predict population-wide implications for delayed diagnosis.^37,38^ Ontario tracks wait time targets for cancer surgery, including time from referral to surgeon appointment, and time from the decision to treat until surgery.^29^ The United Kingdom established the nationwide Two-Week-Wait (TWW) referral program in 2000,^39^ which includes CRC. However, even this large-scale program has not reliably translated into improved outcomes, including survival.^11,40,41,42,43,44,45^ In a meta-analysis of 93 655 patients,^40^ there was no difference in disease stage for those attending the TWW program’s lower GI pathway vs standard referral. Our results suggest time to treatment is closely tied to disease stage at presentation and subsequent triage by physicians, and efforts to shorten this interval further are unlikely to result in meaningful improvements in survival for younger patients with CRC at the population level. Additionally, interventions for postpresentation delays specific to younger adults would be indicated if there was strong evidence they experienced longer times to treatment compared with older adults. In a previous analysis, we compared delay intervals between 6853 adults aged younger than 50 years and 52 144 patients aged 50 to 74 years in Ontario, and showed no significant difference in the overall interval between age groups (adjusted median difference: −0.6 days [95% CI, −4.3 to 3.2 days]).^46^ While there are other benefits of timely treatment, including health care costs, efficient use of resources, and patient distress,^47^ our study (and much of the existing literature^10,13,14^) does not implicate postpresentation delay as a driver of worse outcomes for patients younger than 50 years with CRC.

This study has numerous strengths. We used population-based data and identified patients from a high-quality cancer registry.^48^ To our knowledge, this is the largest study examining delay intervals and survival in patients with CRC younger than 50 years of age.^10^ We investigated prediagnostic intervals, which are underrepresented in the literature,^10^ and we captured time from first presentation until treatment initiation. Our analysis addressed several key methodologic concerns raised by other authors.^17,36,37^ We identified patients who presented emergently and adjusted for this factor in the analysis, stratified models by stage, and repeated the analysis in a subset of lower-urgency patients. Additionally, we used cubic spline regression to allow for nonlinear associations and uncover possible U-shaped associations between the overall interval and mortality.

### Limitations

There are limitations to this study. We identified the date of first presentation using a complex algorithm based on administrative and billing codes specifically developed for Ontario databases.^20^ While it has been used in CRC, breast cancer, oral cancer, pancreatic cancer, and we have previously described its application to young patients with CRC, it has only been directly compared against patient charts for oral cancer.^9,20,21,23^ Our study does not capture the interval between symptom onset and presentation,^37^ and it is possible young adults have delays to presentation that contribute to worse outcomes. It has been recognized that delays in cancer care can also be related to health behavior and psychological factors such as patient knowledge, reluctance to seek help, fear or denial, and financial concerns.^49,50,51^ We were unable to incorporate these factors in our analysis.

## Conclusions

This large, population-based cohort study did not find adverse survival with longer times from presentation to treatment among patients with CRC aged younger than 50 years. Postpresentation delays do not appear to drive advanced disease and poor outcomes in young adults.

## Notes

Supplement 1. **eTable 1.** Using Unique Encrypted ICES Keys, Cohort Data Were Assembled by Linking 15 Separate Data Sources Housed at ICES, Representing Various Aspects of the Publicly Funding Healthcare System in Ontario **eFigure 1.** Cohort Creation Figure for Adults Aged 15-49 Diagnosed With Colorectal Cancer in Ontario From 2007 to 2018 **eTable 2.** Covariate Definitions and Outcome Definitions **eFigure 2.** Time From Presentation to Treatment (Overall Interval) Stratified by Stage for a Cohort of Younger Patients With Colorectal Cancer **eFigure 3.** Additional Descriptive Statistics for Survival **eFigure 4.** Restricted Cubic Spline Regression Demonstrating Univariate Relationship Between Increasing Time From Presentation to Treatment (Overall Interval) and Cause-Specific Survival **eTable 3.** Survival Models Stratified by Stage **eFigure 5.** Spline Regression in Subset of Lower Urgency Patients

Supplement 2.
Data Sharing Statement
