# Supplementary material for: Analysis of Time to Treatment and Survival Among Adults Younger Than 50 Years of Age With Colorectal Cancer in Canada
Source: JAMA Netw Open. 2023 Aug 3;6(8):e2327109. doi: 10.1001/jamanetworkopen.2023.27109 (PMC10401304; doi:10.1001/jamanetworkopen.2023.27109)
Supplement: Supplement 1. — eTable 1. Using Unique Encrypted ICES Keys, Cohort Data Were Assembled by Linking 15 Separate Data Sources Housed at ICES, Representing Various Aspects of the Publicly Funding Healthcare System in Ontario eFigure 1. Cohort Creation Figure for Adults Aged 15-49 Diagnosed With Colorectal Cancer in Ontario From 2007 to 2018 eTable 2. Covariate Definitions and Outcome Definitions eFigure 2. Time From Presentation to Treatment (Overall Interval) Stratified by Stage for a Cohort of Younger Patients With Colorectal Cancer eFigure 3. Additional Descriptive Statistics for Survival eFigure 4. Restricted Cubic Spline Regression Demonstrating Univariate Relationship Between Increasing Time From Presentation to Treatment (Overall Interval) and Cause-Specific Survival eTable 3. Survival Models Stratified by Stage eFigure 5. Spline Regression in Subset of Lower Urgency Patients [file jamanetwopen-e2327109-s001.pdf]

Supplemental Online Content

Castelo M, Paszat L, Hansen BE, et al. Analysis of time to treatment and survival among adults younger than 50 years of age with colorectal cancer in Canada. *JAMA Netw Open.* 2023;6(8):e2327109. doi:10.1001/jamanetworkopen.2023.27109

eTable 1. Using Unique Encrypted ICES Keys, Cohort Data Were Assembled by Linking 15 Separate Data Sources Housed at ICES, Representing Various Aspects of the Publicly Funding Healthcare System in Ontario

eFigure 1. Cohort Creation Figure for Adults Aged 15-49 Diagnosed With Colorectal Cancer in Ontario From 2007 to 2018

eTable 2. Covariate Definitions and Outcome Definitions

eFigure 2. Time From Presentation to Treatment (Overall Interval) Stratified by Stage for a Cohort of Younger Patients With Colorectal Cancer

eFigure 3. Additional Descriptive Statistics for Survival

eFigure 4. Restricted Cubic Spline Regression Demonstrating Univariate Relationship Between Increasing Time From Presentation to Treatment (Overall Interval) and Cause-Specific Survival

eTable 3. Survival Models Stratified by Stage

eFigure 5. Spline Regression in Subset of Lower Urgency Patients

This supplemental material has been provided by the authors to give readers additional information about their work.

© 2023 Castelo M et al. *JAMA Network Open.*

eTable 1. Using unique encrypted ICES keys, cohort data were assembled by linking 15 separate data sources housed at ICES, representing various aspects of the publicly funding healthcare system in Ontario.

Data source	Full name	Description
RPDB	Registered Persons Database	Patient characteristics and vital status
OCR	Ontario Cancer Registry	Cohort identification, tumor characteristics
NACRS	National Ambulatory Care Reporting System	Emergency and ambulatory visits
OCCC	Ontario Crohn's and Colitis Cohort	Identification of inflammatory bowel disease patients
NDFP	New Drug Funding Program	Chemotherapy administration
CAPE	Client Agency Program Enrollment	Primary care physician identification
OHIP	Ontario Health Insurance Plan	Delivery of care and physician interaction
CIHI-DAD	Canadian Institute for Health Information Discharge Abstract Database	Delivery of care and physician interaction
CIHI-SDS	Canadian Institute for Health Information Same Day Surgery Database	Delivery of care and physician interaction
ORGD	Office of the Registrar General Database	Cause of death
IPDB	ICES Physician Database	Physician characteristics
ALR	Cancer Activity Level Reporting	Cancer treatment
Census	Ontario Census	Socioeconomic status
LHIN	Local Health Integration Network	Geographic data
INST	Institute Information System	Hospital and health care institution information

© 2023 Castelo M et al. *JAMA Network Open.*

eFigure 1. Cohort creation figure for adults aged 15-49 diagnosed with colorectal cancer in Ontario from 2007 to 2018.

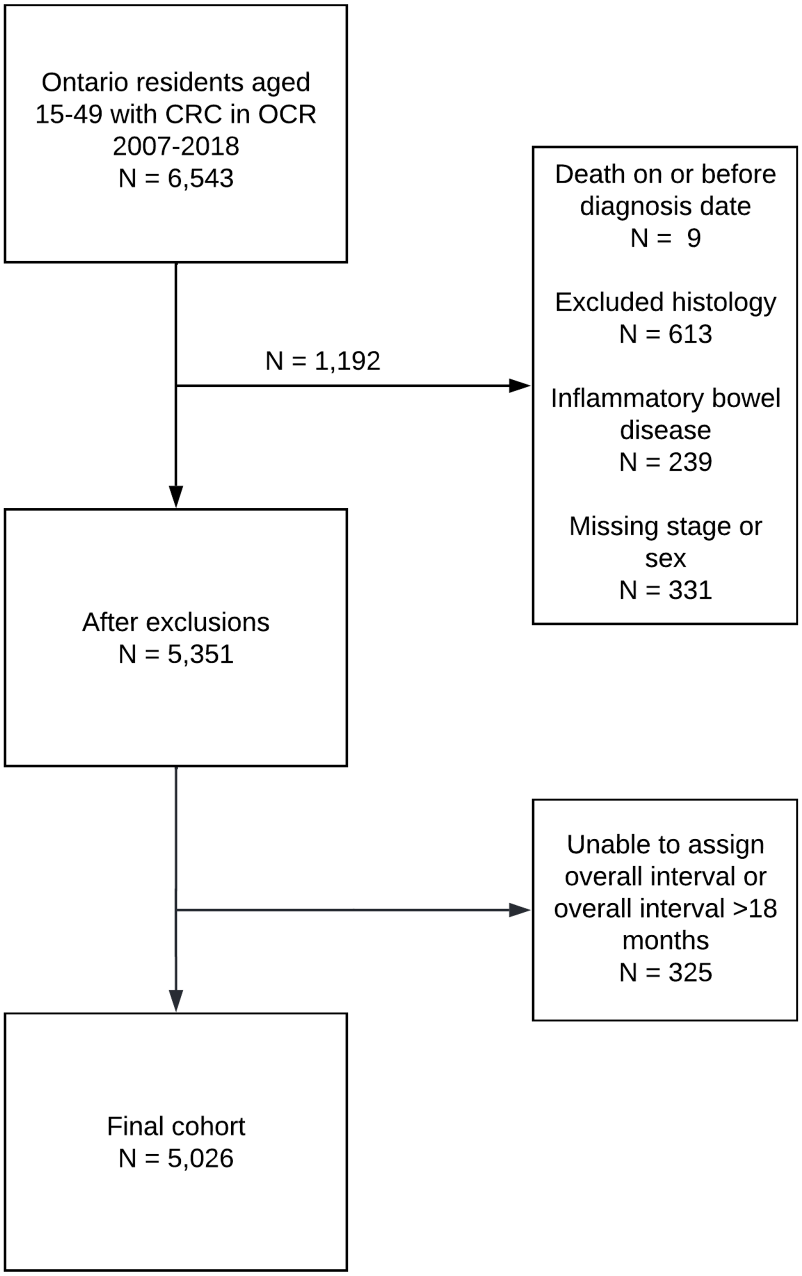

© 2023 Castelo M et al. *JAMA Network Open.*

eTable 2. Covariate definitions and outcome definitions

Variable	Categories	Definitions
Colorectal cancer		International Classification of Diseases version 10 [ICD-10] C18^ - C20^, excluding C18.1 (Appendix)
Inflammatory bowel disease		Ever included in the Ontario Crohn's and Colitis Cohort or the occurrence of ICD-9-CM 555, 555.0-555.9, 556, 556.0-556.9 or ICD-10-CM K500, K501, K508-K515 during an emergency room visit/hospitalization within 5 years prior to the CRC diagnosis date
Histology	Adenocarcinoma/no special type	ICD-O-3 codes 81403, 82633, 82103, 8003, 82613, 80103, 82553, 82113, 80203, 82623, 82133, 83103, 82603, 81433, 85003, 89403, 83803, 83233, 85703, 81406, 82153, 82213, 82443, 82903
	Mucinuous adenocarcinoma	ICD-O-3 codes 84803, 84813
	Other	ICD-O-3 codes 84903, 85103, 85743, 82403, 82463, 80003, 80413, 80703, 81203, 81423, 81443, 81483, 82013, 82703, 83123, 83203, 83373, 84413, 84603, 84703, 85073, 85233, 85303, 85503
Symptomatology	GI symptoms	ICD-10 codes J860, K20, K210, K219, K220, K221, K2210, K2211, K2212, K2213, K2214, K2215, K2216, K2217, K2219, K222, K223, K224, K225, K226, K227, K228, K229, K250, K251, K252, K253, K254, K255, K256, K257, K259, K260, K261, K262, K263, K264, K265, K266, K267, K269, K290, K291, K292, K293, K294, K295, K296, K297, K298, K299, K310, K311, K312, K313, K314, K315, K316, K317, K318, K3180, K3181, K3188, K319, K522, K528, K529, K8000, K8001, K8010, K8011, K8020, K8021, K8030, K8031, K8040, K8041, K8050, K8051, K8080, K8081, K920, K921, K9146, K9160, K9161, K9162, K9169, R066, R100, R1010, R1011, R1012, R1019, R102, R1030, R1031, R1032, R1039, R104, R110, R111, R112, R113, R118, R12, R130, R132, R138, R17, R18, R190, R191, R192, R198, R630, A09, A090, A099, K350, K351, K352, K353, K358, K359, K500, K501, K508, K509, K510, K511, K512, K513, K514, K515, K518, K519, K552, K5520, K5521, K560, K561, K562, K563, K564, K565, K566, K567, K570, K571, K572, K573, K574, K575, K578, K579, K580, K589, K590, K591, K592, K593, K594, K598, K599, K600, K601, K602, K603, K604, K605, K620, K621, K622, K623, K624, K625, K626, K627, K628, K629, K630, K631, K632, K633, K634, K635, K638, K6388, K639, K640, K6410, K6411, K6420, K6421, K6430, K6431, K644, K645, K648, K649, K650, K658, K659, K670, K671, K672, K673, K678, K910, K911, K912, K913, K9140, K9141, K9142, K9143, K9144, K9145, K9149, K915, K918, K919, R14, R15, R194, R195, R1950, R1958
		OHIP diagnostic codes 530, 531, 532, 535, 536, 537, 574, 787, 009, 455, 540, 555, 556, 560, 562, 564, 565, 567, 569
	Anemia	ICD-10 codes D500, D501, D508, D509, D510, D511, D512, D513, D518, D519, D520, D521, D528, D529, D530, D531, D532, D538, D539, D560, D561, D562, D563, D564
		OHIP diagnostic codes 280, 281, 284, 285
	None/not determined	None of the above codes identified
Initial imaging performed	Cross-sectional	CCI codes 3NM20VA, 3NM20WC, 3NM20WA, 3NM20WE, 3OT20VA, 3OT20WC, 3OT20WA, 3OT20WE, 3ER20VA, 3ER20WC, 3ER20WA, 3ER20WE, 3GY20VA, 3GY20WC, 3GY20WA, 3GY20WE, 3SC20VA, 3SC20WC, 3SC20WA, 3SC20WE, 3OT40VA, 3OT40WC, 3OT40WA, 3OT40WE
		OHIP billing codes X231, X232, X233, X234, X409, X410, X400, X402, X406, X407, X415, X451, X455, X461, X465

© 2023 Castelo M et al. *JAMA Network Open.*

Variable	Categories	Definitions
	Non cross-sectional	CCI codes 3OT30DA, 3OT30DB, 3OT30DC, 3OT30DD, 3OT30DG, 3OT30HA, 3OT30LA, 3NQ10VN, 3NZ10VN, 3NM10VN, 3NK10VV, 3NK10VN, 3NK10WG, 3NK10WX, 3NA10VN, 3NA10WG, 3NL10VN, 3OT10VA, 3OT10VH, 3OT10VZ, 3OT10WG, 3OT10WX, 3OT12VA
		OHIP billing codes J128, J135, J428, J435, X100, X101, X103, X104, X112, X113, X197
	No abdominal imaging	None of the above codes identified

ICD-10 - International Classification of Diseases version 10, ICD-O-3 - International Classification of Diseases Oncology version 3, OHIP - Ontario Health Insurance Plan, CCI - Canadian Classification of Health Interventions, CRC - Colorectal cancer, GI - Gastrointestinal, LHIN - Local Health Integration Network

© 2023 Castelo M et al. *JAMA Network Open.*

eFigure 2. Time from presentation to treatment (overall interval) stratified by stage for a cohort of younger patients with colorectal cancer. Whiskers extend from the hinge to the largest/smallest value no further than 1.5*IQR from the hinge. Outliers are suppressed for improved clarity.

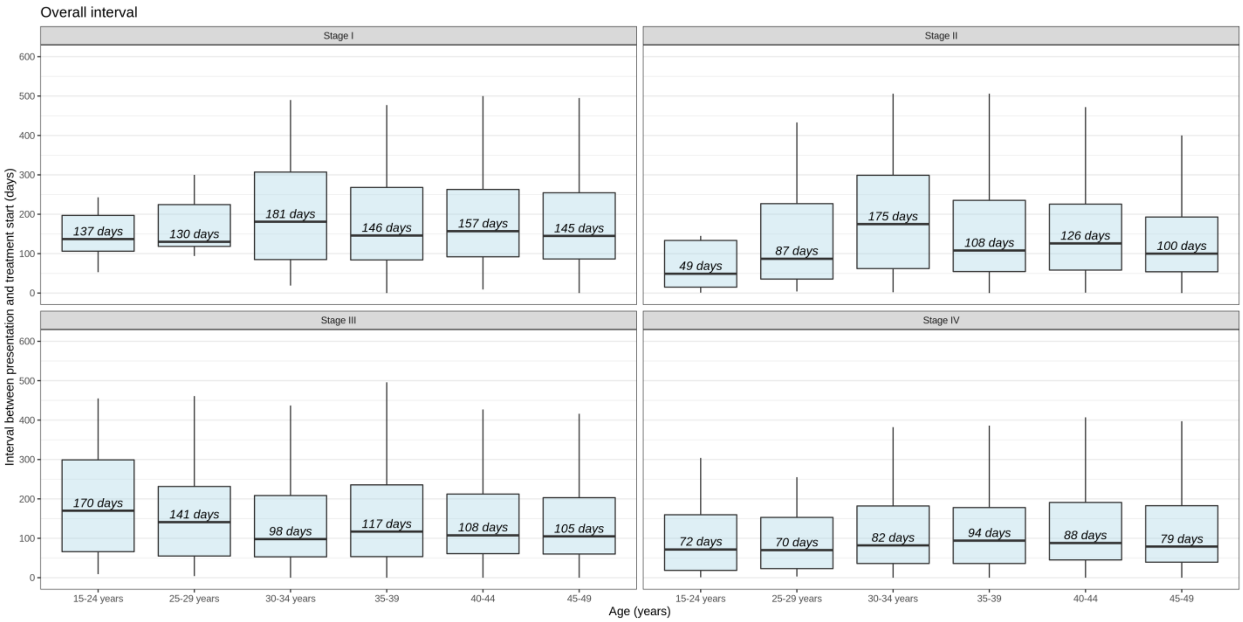

© 2023 Castelo M et al. *JAMA Network Open.*

eFigure 3. Additional descriptive statistics for survival.

Cumulative incidence function demonstrating deaths due to colorectal cancer and other causes among younger adults with colorectal cancer.

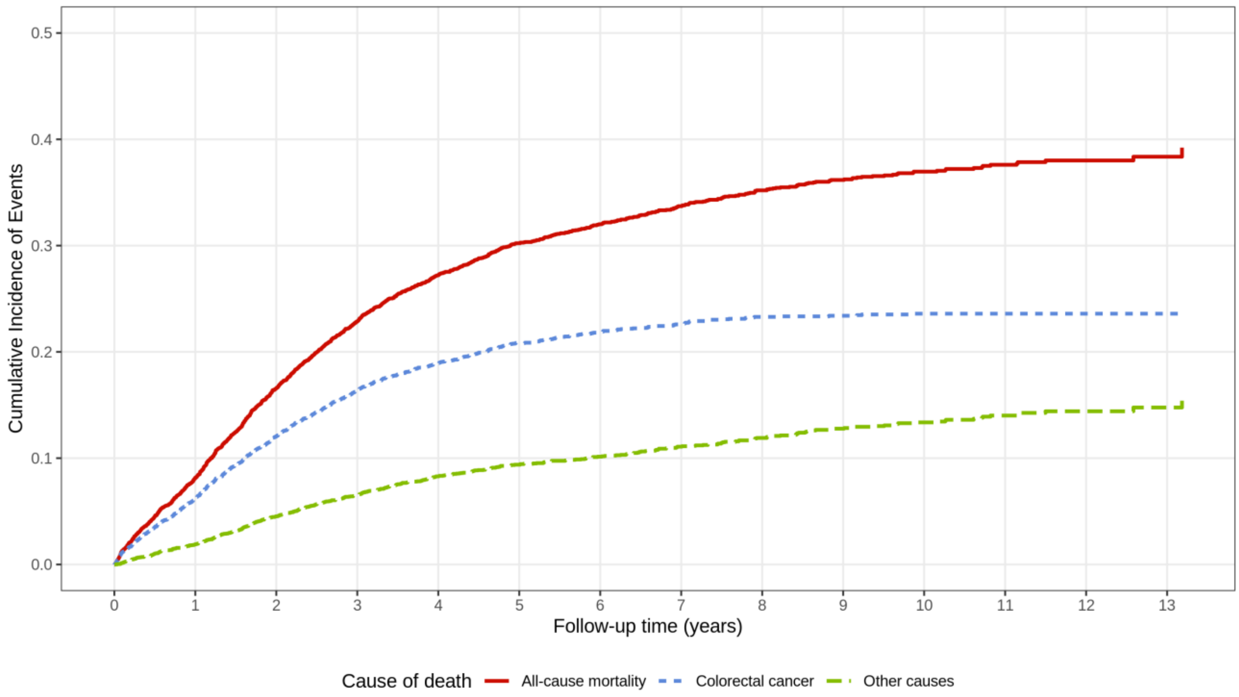

5-year and 10-year overall and cause-specific survival for colorectal cancer patients <50 years by stage.

**Stage**	**Overall survival (95% confidence interval)**		**Cause-specific survival (95% confidence interval)**	
	**5-year**	**10-year**	**5-year**	**10-year**
All stages	69.8% (68.4-71.1%)	63.0% (61.5-64.6%)	78.2% (77.0-79.4%)	75.0% (73.7-76.4%)
Stage I	95.5% (93.9-97.1%)	90.4% (87.6-93.3%)	98% (96.9-99.1%)	97.8% (96.6-98.9%)
Stage II	90.9%| (89.1-92.8%)	86.7% (84.3-89.1%)	94.3% (92.8-95.8%)	92.4% (90.6-94.2%)
Stage III	79.2% (77.2-81.1%)	70.7% (68.1-73.2%)	85.6% (83.9-87.3%)	81.1% (78.9-83.2%)

© 2023 Castelo M et al. *JAMA Network Open.*

Stage	Overall survival (95% confidence interval)		Cause-specific survival (95% confidence interval)	
	5-year	10-year	5-year	10-year
Stage IV	20.5% (18-23%)	12.4% (10-14.8%)	33.1% (29.7-36.4%)	27% (23.3-30.7%)

eFigure 4. Restricted cubic spline regression demonstrating univariate relationship between increasing time from presentation to treatment (overall interval) and cause-specific survival. Hazard ratios shown on a log scale.

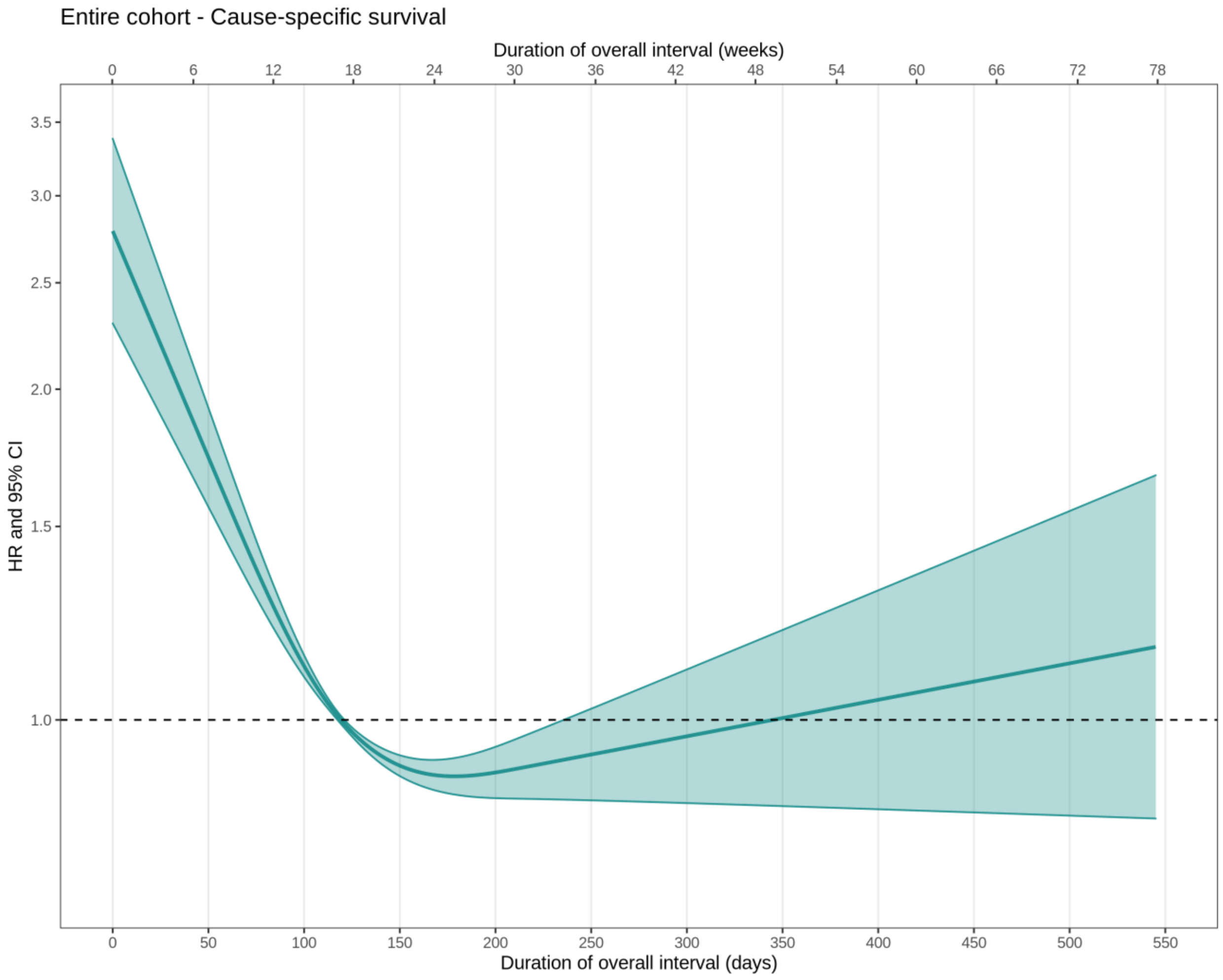

© 2023 Castelo M et al. *JAMA Network Open.*

eTable 3. Survival models stratified by stage.

Adjusted Cox proportional hazards model (HR and 95% CI) showing effect of increasing overall interval on overall survival in the entire patient cohort, stratified by stage of disease.

Length of overall interval	Stage I	Stage II	Stage III	Stage IV
0 to less than 6 weeks	1.05 (0.29, 3.84)	1.26 (0.59, 2.70)	1.39 (1.01, 1.92)	1.35 (1.09, 1.67)
6 to less than 12 weeks	0.82 (0.32, 2.13)	1.21 (0.62, 2.39)	0.95 (0.70, 1.29)	1.23 (1.00, 1.53)
**12 to less than 18 weeks**	**Reference**	**Reference**	**Reference**	**Reference**
18 to less than 24 weeks	0.84 (0.31, 2.28)	1.25 (0.57, 2.75)	0.90 (0.62, 1.30)	1.02 (0.76, 1.39)
24 to less than 30 weeks	0.95 (0.34, 2.63)	1.15 (0.49, 2.71)	0.76 (0.48, 1.21)	0.84 (0.61, 1.15)
30 to less than 36 weeks	0.70 (0.20, 2.51)	1.53 (0.57, 4.09)	0.79 (0.50, 1.23)	1.01(0.72, 1.40)
36+ weeks	0.85 (0.38, 1.89)	1.56 (0.80, 3.05)	0.86 (0.62 ,1.18)	0.97 (0.76, 1.23)

Adjusted for sex, age, number of major ADGs, symptomatology, ON-Marg score, emergency presentation, and cancer site

Adjusted Cox proportional hazards model (HR and 95% CI) showing effect of increasing overall interval on cause-specific survival in the entire patient cohort, stratified by stage of disease.

Length of overall interval	Stage I	Stage II	Stage III	Stage IV
0 to less than 6 weeks	1.05 (0.29, 3.83)	1.26 (0.59, 2.70)	1.40 (1.01, 1.92)	1.35 (1.09, 1.67)
6 to less than 12 weeks	0.82 (0.32, 2.14)	1.21 (0.62, 2.39)	0.95 (0.70, 1.29)	1.23 (1.00, 1.53)
**12 to less than 18 weeks**	**Reference**	**Reference**	**Reference**	**Reference**
18 to less than 24 weeks	0.84 (0.31, 2.28)	1.25 (0.57, 2.76)	0.90 (0.62, 1.30)	1.04 (0.77, 1.41)
24 to less than 30 weeks	0.94 (0.34, 2.61)	1.15 (0.49, 2.72)	0.77 (0.48, 1.22)	0.85 (0.62, 1.16)
30 to less than 36 weeks	0.69 (0.19, 2.48)	1.52 (0.57, 4.07)	0.79 (0.50, 1.23)	1.01 (0.73, 1.41)
36+ weeks	0.85 (0.38, 1.89)	1.57 (0.80, 3.05)	0.86 (0.62, 1.19)	0.97 (0.76, 1.22)

Adjusted for sex, age, number of major ADGs, symptomatology, ON-Marg score, emergency presentation, and cancer site

© 2023 Castelo M et al. *JAMA Network Open.*

eFigure 5. Spline regression in subset of lower urgency patients.

Restricted cubic spline regression demonstrating univariate relationship between increasing time from presentation to treatment (overall interval) and overall survival. Hazard ratios shown on a log scale. A) In subset of lower urgency patients B) Stratified by stage of colorectal cancer among subset of lower urgency patients.

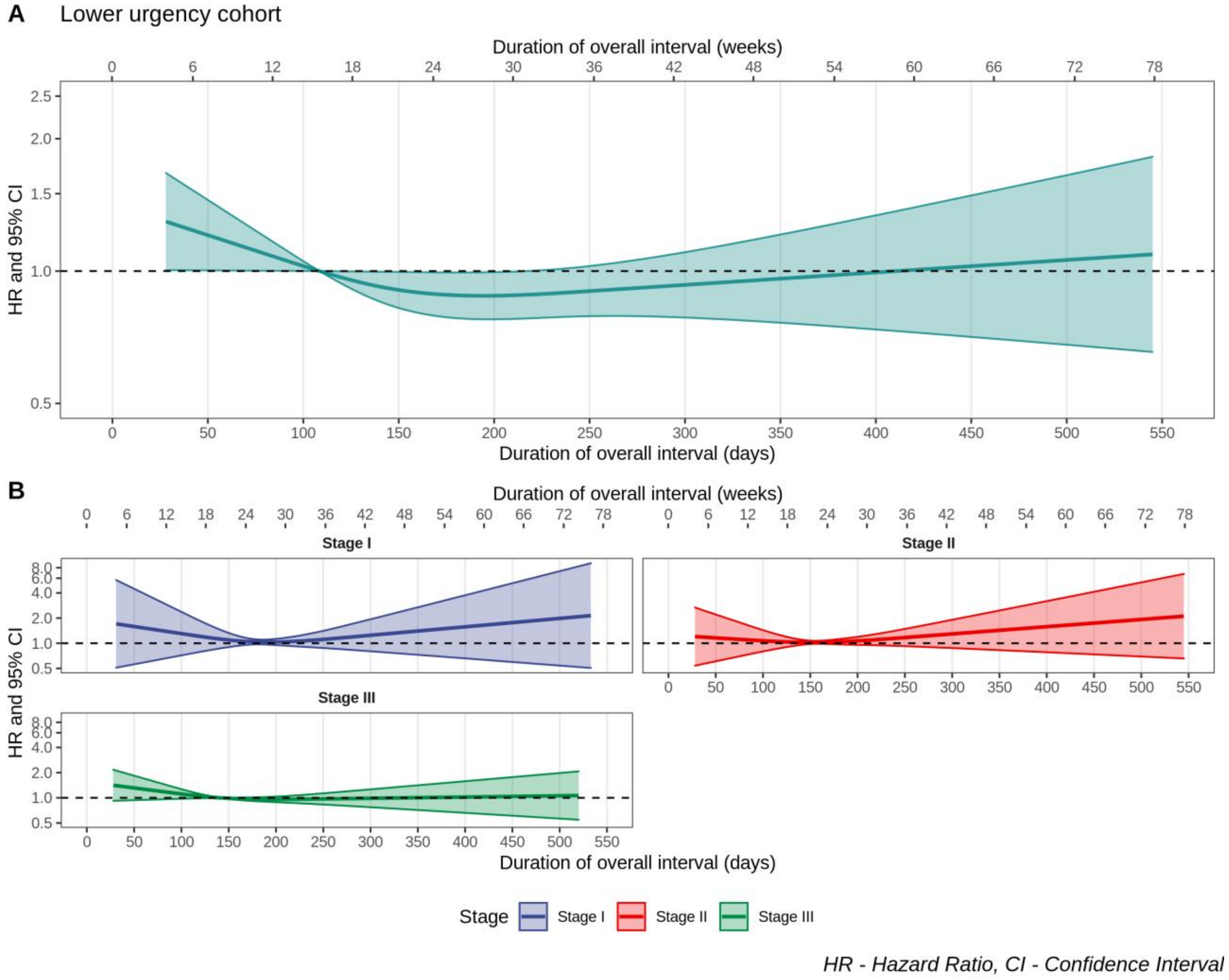

© 2023 Castelo M et al. *JAMA Network Open.*

Restricted cubic spline regression demonstrating univariate relationship between increasing time from presentation to treatment (overall interval) and cause-specific survival in subset of lower urgency patients. Hazard ratios shown on a log scale.

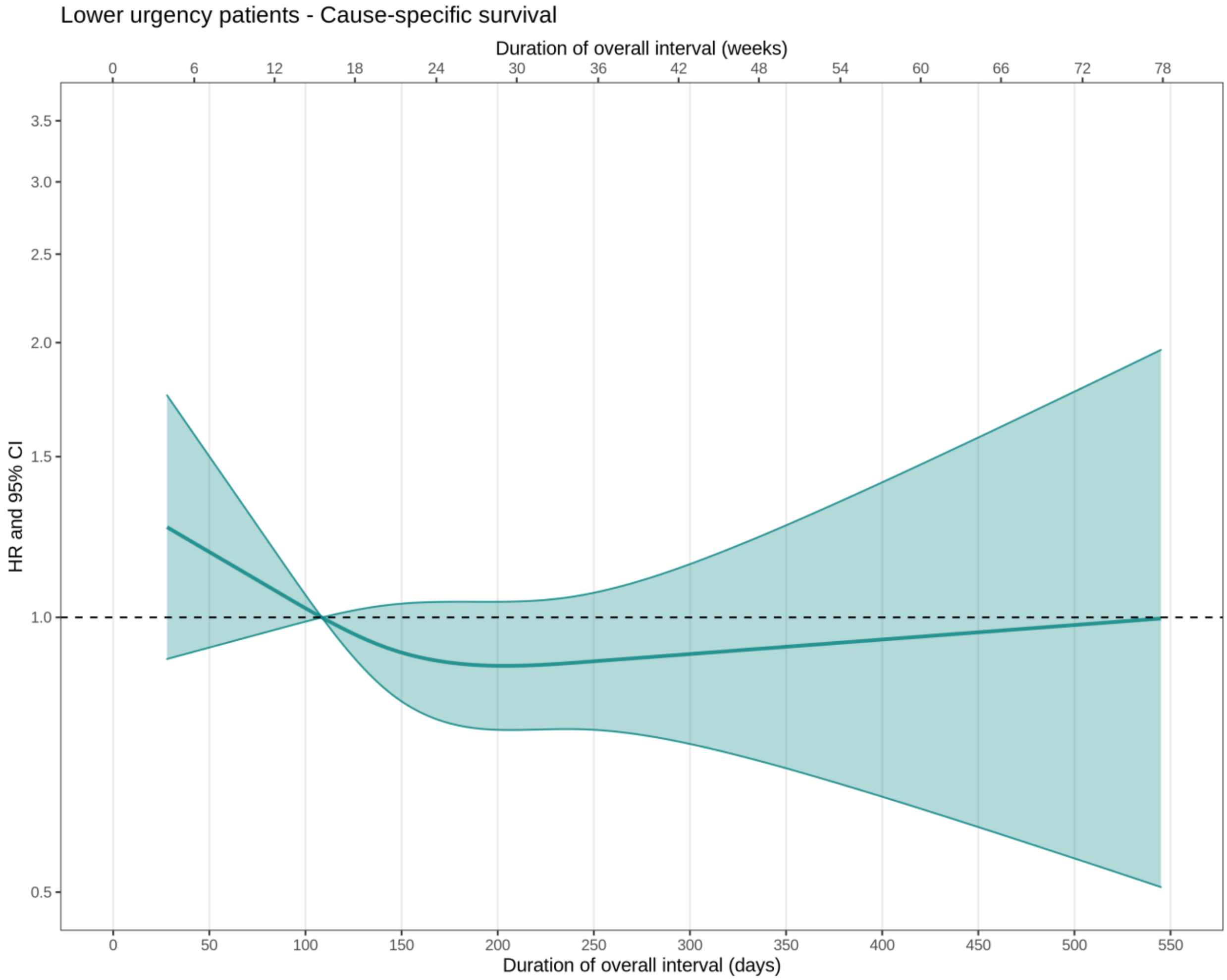

© 2023 Castelo M et al. *JAMA Network Open.*
